# Pre-positioning an evaluation of cash assistance programming in an acute emergency: strategies and lessons learned from a study in Raqqa Governorate, Syria

**DOI:** 10.1186/s13031-021-00340-1

**Published:** 2021-03-01

**Authors:** Kathryn Falb, Jeannie Annan

**Affiliations:** 1 Airbel Impact Lab, International Rescue Committee, 1730 M St NW, Suite 505, Washington DC, 20036 USA; 2 Airbel Impact Lab, International Rescue Committee, 122 E 42nd St, New York City, NY 10168 USA

**Keywords:** Syria, Internally displaced people, Intimate partner violence, Sexual exploitation, Humanitarian, Economic empowerment, Cash transfer

## Abstract

**Background:**

Conducting ethical and rigorous research to measure the effectiveness of humanitarian programs is urgently needed given the global level of displacement and conflict, yet traditional approaches to evaluation research may be too slow and disruptive for acute humanitarian settings. The current case study utilizes an experience of implementing a mixed methods evaluation conducted between March–August 2018 in northern Raqqa Governorate, Syria. The key research objectives were to examine the influence of an unconditional, three-month cash transfer program on household basic needs and women’s wellbeing, including experiences of violence. This setting was selected for the research as it shared many aspects of an acute emergency within a protracted conflict given its recent opening of access to humanitarian aid programming following the withdrawal of ISIS as well as influxes of internally displaced persons fleeing airstrikes and fighting in Raqqa City in late 2017.

**Discussion:**

The current case study was scientifically important as the use of cash assistance in emergencies has increased exponentially in recent years. Yet, little is still known about how cash assistance designed to help households meet their basic needs may also influence women’s overall wellbeing in the home. Challenges of conducting the research included selecting an emergency site appropriate for research, implementing an evaluation that would not delay or disrupt critical cash assistance programming, and measurement of sensitive violence against women outcomes. Four strategies were identified to meet the challenges of conducting an evaluation in such a setting, which included: (1) developing clear decision-making criteria for assessing feasibility; (2) frontloading processes to reduce time lag in launching research; (3) integrating the research approach within programming; and (4) closely collaborating with practitioners throughout the study, especially for research on sensitive topics like violence against women. Conclusions

Advance consideration of these factors through a pre-positioning process will allow for timely, ethical, and rigorous research to be implemented in the immediate aftermath of a crisis. Such studies should be prioritized to ensure the highest effectiveness and efficiency of humanitarian aid for populations grappling with acute emergencies.

## Background

In 2018, there were over 68.5 million displaced people who have been affected by armed conflicts, natural disasters, public health emergencies, or other persecution, reaching some of the highest numbers of displacement the world has ever witnessed. This equates to one person being forcibly displaced every 2 s [1]. Nonetheless, over a 34 year period ending in 2014, only 345 studies were identified in a recent systematic review that evaluated public health interventions in humanitarian crises – and most were of variable quality [2]. Moreover, even fewer evaluations have been conducted in acute emergency settings, which arise from natural disasters or the eruption or exacerbation of conflict.

While consensus has largely been reached about the importance of conducting rigorous needs assessments in acute emergencies, equivalent support for evaluations has lagged. This may be because the way research is traditionally done is too slow and disruptive for these settings and, at times, too opaque for practitioners focused on meeting the needs of affected populations. However, without rigorous and ethical evaluation research conducted in acute emergency settings, the humanitarian field, *at best*, may unwittingly divert resources from more effective or efficient programming models to meet the needs of the populations, and, *at worst,* unintentionally create harm or exacerbate risks amongst those affected by conflict and disaster.

In this paper, we demonstrate that it is possible to conduct ethical and rigorous research in acute emergencies and draw on a recent experience of evaluating the potential effect of a cash assistance program on women’s experiences of violence and wellbeing in Raqqa Governorate, Syria, conducted by the International Rescue Committee (IRC), an international humanitarian aid organization. Based on the experience from this study, we outline four necessary strategies researchers can take to prepare for and conduct research in an ethical way that reduces time delays and involves key programmatic actors in the design.

### Humanitarian context in northeastern Syria

Commencing in 2011, the civil war in Syria, during which it is estimated that at least half a million people have been killed [3], catalyzed one of the largest displacements in recent global history to neighboring countries. More than 6 million people were also displaced internally and exposed to extreme violence and deprivation [4]. Raqqa Governorate in Northeast Syria has been one of the most adversely affected geographical areas in the fighting for both internally displaced people residing there as well as the host communities that remained.

Raqqa Governorate, historically one of the more economically disadvantaged parts of the country [5], also faced additional instability and conflict due to the proliferation of Islamic State of Iraq and Syria (ISIS) between 2013 and 2017. ISIS occupied Raqqa City and many villages throughout the governorate during this period, which led to mass atrocities, particularly for women and girls [6]. Further, these areas were often left inaccessible for humanitarian aid organizations to provide adequate programming due to security concerns. The fighting peaked in late 2017, during which most of Raqqa City was destroyed due to airstrikes from a U.S. led coalition, resulting in ISIS largely withdrawing from the area and high levels of internal displacement to northern Raqqa Governorate [7] During the period of the study implementation (March – August 2018) when humanitarian actors gained renewed access to the geographical area, the population in Raqqa Governorate was still reeling from the aftermath of war and ISIS occupation due to the destruction of infrastructure, broken health systems, and limited access to shelter, food, or ability to re-establish livelihoods.

### Research study

Dozens of humanitarian aid organizations and donors alike have made global commitments to increase their use of cash transfers as a key strategy to assist people in the aftermath of an emergency in recent years [8]. For instance, while cash transfers represented only 2.5% of aid in 2015, it accounted for 10% of humanitarian aid in 2016 [9]. Overall, trends point towards the use of cash exponentially increasing in future years as a mechanism to meet the basic needs of the highest numbers of people in the shortest periods of times in acute emergencies. In fact, IRC itself committed to delivering at least 25% of material assistance to populations affected by crises through cash modalities by 2020.

Despite this increased investment in cash assistance in recent years, little is known about how cash influences the household dynamics and experiences of violence amongst women in humanitarian settings, including sexual exploitation or intimate partner violence (IPV). Such investigation is important as Syrian women often experience different forms of violence, including IPV [10] and baseline data from the present study indicated that a staggering one in three women in the sample had experienced IPV in the past 3 months [11]. In largely stable low and middle income countries, a recently completed systematic review of cash transfers, typically delivered as part of long standing social protection schemes, revealed that many cash transfer programs are associated with reductions of IPV; however, a small number of other programs had no or negative effect [12]. Contextual differences, such as pre-existing gender norms, and programmatic differences such as who is targeted within the home (i.e., male versus female), length of cash transfer programming, or complementary programming may influence whether cash positively or negatively influences women’s experiences of IPV [13]. In particular, a key difference between this type of longer term cash programming and humanitarian cash programming is the length in which cash transfers are delivered. Often times, and in the case of Raqqa Governorate, Syria, humanitarian cash assistance is delivered on a monthly basis for a period of 3 months, with a value set based on market assessments, to meet populations’ critical basic needs. Targeting is directed towards the head of household, with no pre-determined specifications for male versus female heads.

Given this overall largely positive, but still mixed evidence on how cash or other forms of economic empowerment [14] may reduce or exacerbate risks for women, alongside the increasing use of cash as a humanitarian intervention, the objective of this study was to better understand the impact of cash transfers in acute emergencies on women’s experiences of violence and exploitation, mental health, and economic wellbeing [15]. The study design through which this case analysis is drawn from was a quantitative pre-post test conducted with 512 women at baseline in March 2018 and 456 at endline in August 2018 (loss to follow up: 10.9%) implemented in three villages in northern Raqqa Governorate [15]. The baseline was delivered in advance of the first cash transfer; the endline survey was administered two-three weeks after the final cash disbursement as no long term impacts of cash were hypothesized. Interviews were delivered female enumerators who were identified, contracted, and trained by the International Rescue Committee in the Arabic language through electronic data collection techniques (additional measurement approaches described below). At endline, qualitative in-depth interviews using a line history technique were also implemented with 40 women selected through a maximum variation sampling methodology based on marital status, age, location, and report of violence at baseline.

## Discussion

### Scientific importance

There have been limited undertakings of mixed methods evaluations of cash based programming on the lives of women in acute emergencies, despite its proliferation as a tool to deliver aid in recent years. For instance, in a recent review of gray literature, around 30 investigations of humanitarian cash programming included elements of gender dynamics and violence; however, most rely on qualitative data and have not been taken place in acute settings [13]. In particular, critical questions remain regarding the potential intended and unintended influences of cash on women within the home. Additionally, the study discussed in this paper took place inside Raqqa Governorate, Syria, which represents one of the first systematic mixed methods data collection efforts inside the highly insecure country in recent years. Findings of this study were presented in policy and practitioner briefs, academic manuscripts, as well as at key policy moments to influence the humanitarian field, including calls for further research into the causal relationship between cash assistance and women’s experiences of violence. Validation workshops were held in which programmatic recommendations were developed with the IRC team in Northeast Syria to incorporate findings into program design. Findings are important for the consideration of cash programming in acute settings and highlights potential areas for inquiry in protracted emergencies.

### Challenges to research

The main challenges that emerged in seeking to answer this critical research question were around *selecting an acute emergency site* in which rigorous research could be conducted, while still ensuring that ethical parameters could be met. In this case, the ability to address ethical concerns were of critical importance given our primary outcomes centered on experiences of violence against women. *Clear decision-making protocols and selection criteria, developed in collaboration with practitioners* was necessary to overcome these challenges; the process of which is described in further detail below.

Additional challenges that emerged when seeking to pre-position a study were ensuring that *research or evaluation would not delay nor disrupt potentially life-saving delivery of humanitarian cash assistance*. The process of frontloading research processes and integrating research design activities alongside program delivery, described in further detail below, were necessary to ensure there were no lag times in cash distributions due to the research study.

Finally, once the study site was selected, additional challenges emerged related to *conducting research on violence against women*, including transactional sexual exploitation and abuse (SEA), and other sensitive outcomes in an insecure setting. While these experiences are stigmatizing globally, these outcomes were particularly so in the Northeastern Syria setting. Additional steps undertaken to address these measurement, ethical and security issues are described in the following section.

### Research strategies

#### Strategy 1. Develop a decision making process with researchers and practitioners that ensures strong partnership and clear selection criteria for research in an acute setting

In the first stage, members of the IRC research and practitioner team developed specific criteria that needed to be met by any study setting in order to conduct an ethical and high-quality evaluation. Consideration of factors related to programming capacities and ethics of violence against women research in potential settings was paramount. Exemplary criteria included: local team capacity to undertake or host research in a setting, minimum services (e.g., case management) available for women participating in the survey who were experiencing violence, confidential space availability for interviews, etc. A scoping process was then conducted to map potential emergencies, such as cyclical natural disasters or expected displacements due to increased conflict.

The first site selected for the study that met the above stated criteria was Sindh Province, Pakistan as annual flooding often necessitated the distribution of cash transfers as emergency response to support families. In anticipation of the cyclical flooding and cash response, surveys were developed, translated, and piloted, along with the development of pre- and post-crisis work plans. However, in 2016, when the research was anticipated to begin, the flooding was not as severe and did not necessitate the large scale distribution of cash. Given other external and operational constraints, the study did not move forward the following year in Pakistan and a scoping and decision-making process was re-launched to select another acute emergency in which to conduct the research.

During this second scoping process, Raqqa Governorate, Syria emerged as a potential site. While the conflict within Syria is protracted in nature as a whole, Raqqa Governorate received an influx of internally displaced people fleeing airstrikes in Raqqa City in 2017. In addition, ISIS had recently withdrawn from villages in the governorate which allowed new access for humanitarian programming. Ultimately, these factors share many similarities with that of an acute emergency setting where IRC would implement cash programming. Further, there was a strong presence from IRC women’s protection and empowerment (WPE) teams such that ethical and safety issues related to violence against women research were able to be appropriately addressed and mitigated.

#### Strategy 2. Frontload processes that can be done in advance to reduce time lag

In the second simultaneous step of prepositioning this evaluation, research designs were developed and assessed for feasibility and the first stage of the ethical review was completed even before the location was finalized in a staged review process. Three main quantitative designs were considered: (1) a randomized controlled trial (RCT); (2) a regression discontinuity design; and (3) a pre-posttest; trade-offs of each were carefully deliberated. Of note, all designs included a robust qualitative component that comprised of in-depth interviews utilizing life line histories with women.

In this case, an RCT was deemed unethical in an acute emergency setting due to the need for a pure control group that did not receive the cash transfer to meet basic needs. Using alternative treatment options for comparisons, such as the distribution of non-food items or vouchers were considered inappropriate as cash transfers, particularly within the Northeast Syria context, are the primary modality in which IRC, and the broader humanitarian community is moving towards due to its effectiveness to meet basic needs and its projected efficiency and timeliness. Thus, the ‘standard of care’ is cash and randomizing potentially less efficient programming was not appropriate. Additional comparisons were considered such as a waitlisted group, but this was not deemed ethical in such an acute humanitarian setting. Utilizing a comparison group which might allow for a phased or staged research approach was also not possible as cash was rolled-out community by community and too few communities targeted in this specific rollout for sufficient statistical power nor was the comparison between cash given one to two months earlier than others a programmatically relevant comparison. Other quasi-experimental designs were assessed (one is further described below), however, they were ultimately deemed not feasible or ethical to answer the research questions. Therefore, a pre-posttest, with qualitative data collection was finally determined to be the most ethical and feasible design, despite limitations to causal inference.

While the study design discussions were ongoing, the research team developed ‘study shells’ for each approach that accounted for logistical constraints, security concerns, and ethical considerations that were submitted to the IRC Institutional Review Board. This allowed the review process to occur in stages where provisional reviews were conducted in advance of an acute emergency to limit any ethical review delays. Once the final emergency setting was selected, modifications to the protocol were quickly submitted for timely review by IRB members who were already familiar with the study to not delay any programmatic implementation or the baseline research. Within the final Northeast Syria context, no functional review board was available; however, relevant authorities provided approval for conducting the study. Further description of the adaptations needed for ethical reviews for research in humanitarian settings can be found in Falb et al., 2019 [16].

#### Strategy 3. Integrate research with the programmatic approach to minimize disruption to needed services

The research and programmatic team worked to integrate the research within the programmatic approach to reduce changes that would need to take place in launching cash transfers in such challenging contexts. Initially, it was thought that a regression discontinuity design (RDD) would allow this integration because of how IRC, and many other organizations, deliver cash programming, and has been used in other cash evaluations [17]. For instance, as part of the targeting approach, community members are screened and receive a vulnerability score from several indicators, assessed on a continuous scale, which determines whether they would be eligible to receive cash programming. Cut-off scores are then used in the vulnerability scale to determine whether a person or household would receive cash programming. Therefore, a natural comparison group would be created through the vulnerability assessments by examining those closely clustered above and below the cut-off within an RDD approach.

However, although this research design would have been highly integrated with the programmatic approach with minimal disruption, the vulnerability scores were so high in Raqqa Governorate that there were not enough potential participants in this setting to form an appropriately sized comparison group who would not receive cash for statistical analyses. Although the RDD did not move forward, this thinking about programmatic integration will nonetheless be useful for future designs and the principle of integrating programmatic designs is an important one for acute settings.

#### Strategy 4. Work closely and equitably with local practitioners throughout the study, including making decisions about design, implementation, and monitoring of risks in an insecure setting

Violence against women is a highly sensitive issue in which additional research precautions must be undertaken. Within this study, Syrian practitioners leading IRC’s WPE programming were consulted throughout the research design process. They provided in-depth training to data collectors on gender-based violence and fed into the development and translations of measures, as well as interpretation of results. Practitioners also provided guidance on where to conduct interviews – at times, in confidential offices within their current buildings where they provided programming or elsewhere, including a rented hotel for the purposes of the study where privacy could be ensured. Practitioners also provided guidance on referral pathways for participants, such that after survey completion, all women were given information about services available if they were experiencing violence. Given there is limited evidence on the potential positive or negative influence of cash on experiences of violence in humanitarian settings practitioner guidance on mitigating any potential risks for women was particularly important.

Additionally, close engagement with practitioner colleagues on key study decisions was critical to the success of this study overall. For instance, one of the original main objectives of the study was to determine whether cash transfers had any potential influence on women’s transactional sexual exploitation and abuse (SEA) as women are often forced to engage in this activity in humanitarian emergencies in order to meet their and their families’ basic needs [18]. While the research team engaged closely with local authorities for permission to conduct the study, an increase in research scrutiny was reported by IRC access/security teams between baseline and endline. Given this increased risk, a recommendation to remove the measurement of SEA at endline was given and adhered to. While the study could no longer make inferences about whether cash influenced this key outcome, it was critical to engage with practitioner team members to ensure an ethical decision was made and that no additional risks were placed on women.

## Conclusion

The process of prepositioning an evaluation for an acute emergency setting is not to be underestimated; this example took place over the course of 3 years and successful implementation of the study was only possible through long-term, flexible donor funding. The study team faced a number of challenges, including finding an appropriate site with an acute emergency, designing a study that did not interfere with the implementation of humanitarian programming, and obstacles in measuring sensitive outcomes. Despite challenges faced in this example, research in acute emergencies is possible and should continue to strive for the inclusion of counterfactuals to improve causal inference of humanitarian programming, using ethically chosen experimental or quasi-experimental designs. Assessing the feasibility of grafting research onto organic program rollout in order to implement a waitlisted or staged research design may be a promising avenue if ethical considerations are met. In addition, research efforts must be conducted in close and equitable partnership with practitioners in order to ensure that ethical and security considerations are appropriately addressed. Research must also be grounded in and guided by the realities and voices of people affected by conflict and crises through qualitative research and other participatory approaches. Advance consideration of these factors through a pre-positioning process allows for timely, ethical, and rigorous research to be implemented in the immediate aftermath of a crisis. Such studies should be prioritized to ensure the highest effectiveness and efficiency of humanitarian aid for populations grappling with acute emergencies.

## Data Availability

The datasets generated and/or analyzed during the current study are not publicly available due to the sensitivity of the data collected, but are available from the corresponding author on reasonable request.

## Abbreviations

GBV: Gender based violence
IPV: Intimate partner violence
IRC: International Rescue Committee
ISIS: Islamic State of Iraq and Syria
IRB: Institutional Review Board
RCT: Randomized Controlled Trial
RDD: Regression Discontinuity Design
SEA: Sexual exploitation and abuse
WPE: Women’s Protection & Empowerment
